# P-1539. Staphylococcus aureus clinical isolates display staphylothrombin activity patterns corresponding to sequence type

**DOI:** 10.1093/ofid/ofaf695.1720

**Published:** 2026-01-11

**Authors:** Jordan Kim, Brooke M Talbot, Sarah W Satola, Michael Z David, Timothy D Read, Gregory L Damhorst

**Affiliations:** Emory University, Atlanta, GA; Emory University, Atlanta, GA; Emory University School of Medicine, Division of Infectious Diseases, Atlanta, Georgia; University of Pennsylvania Perelman School of Medicine, Philadelphia, Pennsylvania; Emory University School of Medicine, Atlanta, Georgia; Emory University, Atlanta, GA

## Abstract

**Background:**

*Staphylococcus aureus* is a leading cause of bacterial infections globally, which range from relatively benign tissue and skin infections to severe bloodstream and intravascular infections. A prominent virulence and survival factor of many *Staphylococcus* species is the ability to form thrombi in blood via the conversion of fibrinogen to fibrin using prothrombin-activating proteins. We previously created an assay to measure this phenomenon in vitro. Our objective here was to compare fibrin formation phenotypes across common *S. aureus* multilocus sequence types (STs) using laboratory and clinical strains.

**Table 1:**
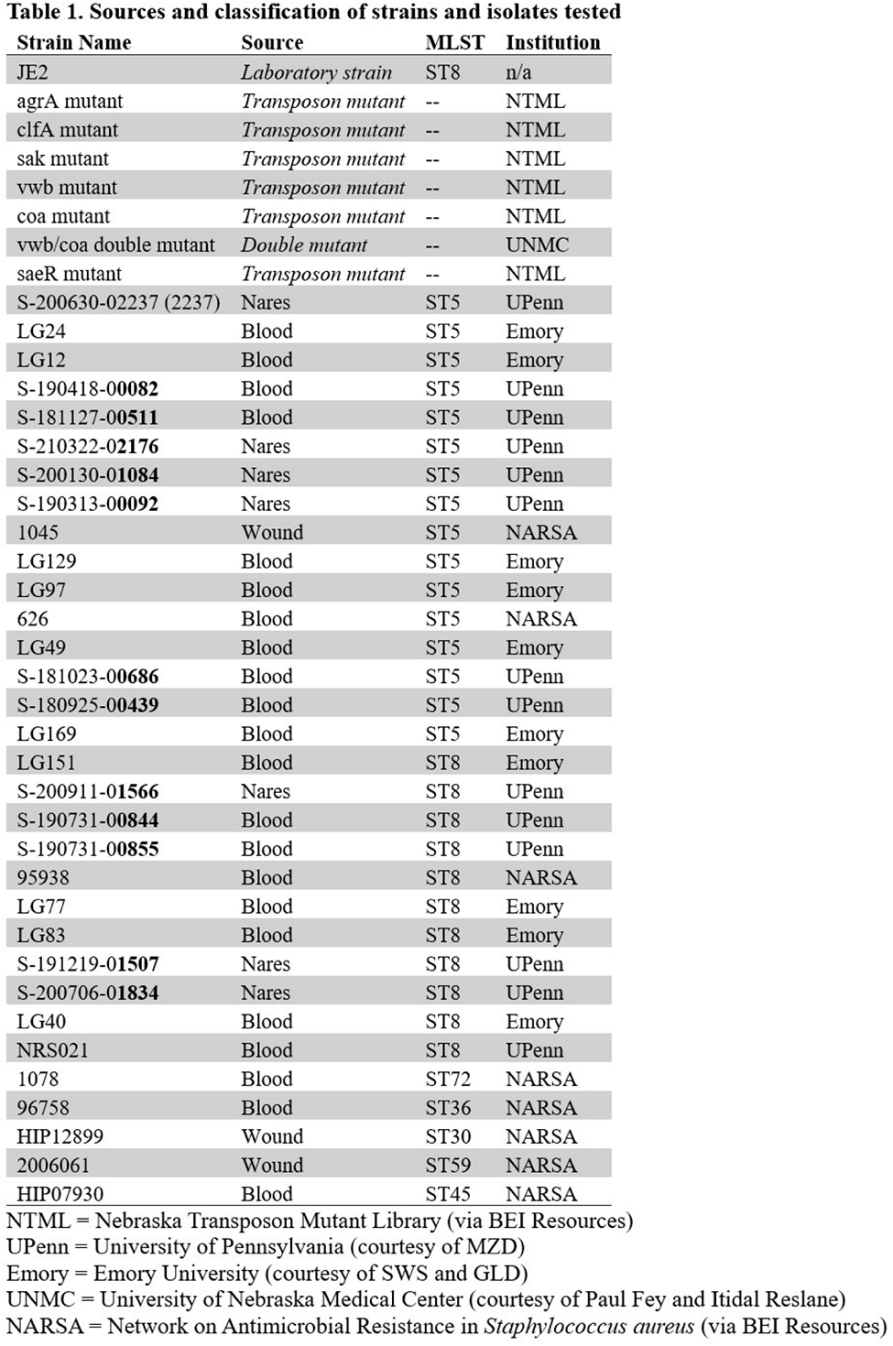
Staphylococcus aureus strains used in Staphylothrombin activity and Growth fitness Assay (STGA) List of all 32 S. aureus strains used in STGA experiments including the following: Source of isolate, MLST, and the institution the isolate was received from.

**Figure 1:**
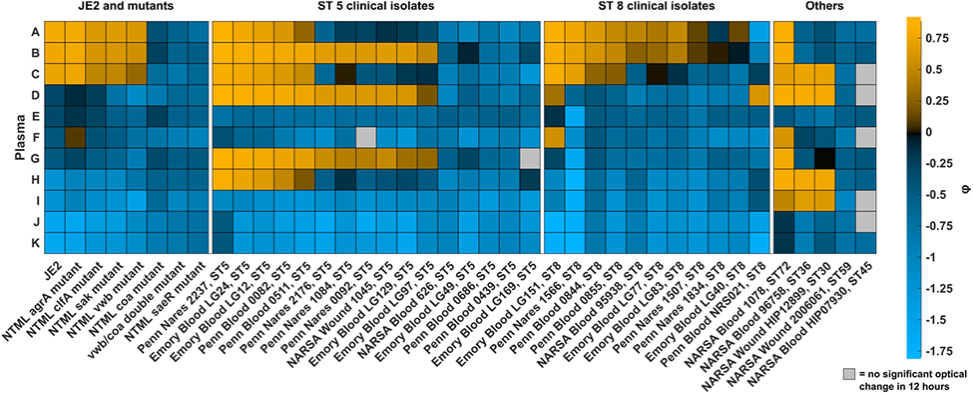
Heatmap of plasma specimens against different Staphylococcus aureus strain supernatants Staphylothrombin activity and growth fitness assay (STGA) results for JE2 mutants and 32 clinical isolates with 11 unique plasma specimens showed within-ST patterns suggesting that underlying bacterial genetic factors may determine staphylothrombin activity phenotypes in human hosts. Blue squares represent a smaller phi value (lower reactivity) while yellow squares represent a higher phi value (higher reactivity).

**Methods:**

We used our novel, 96-well plate-based Staphylothrombin activity and Growth fitness Assay (STGA) to characterize a panel of 11 plasma specimens and culture supernatant from *S. aureus* JE2, 7 mutants (JE2 background), 16 ST5 clinical isolates, 11 ST8 clinical isolates, and 5 additional NARSA reference strains of other STs (Table 1). The STGA quantifies optical changes from fibrin formation in plasma relative to growth of bacteria in broth (signified by φ) when culture supernatant, which contains secreted coagulases, is introduced. φ >0 suggests avid fibrin formation while φ< 0 suggests limited fibrin formation.

**Results:**

Human plasma specimens from different donors were designated A-K based on average φ with the JE2 reference strain. Three plasma specimens (A, B, C) demonstrated avid fibrin formation with JE2 and loss of phenotype as expected in *coa* and *SaeR* JE2 mutants (Figure 1). Most ST8 clinical isolates demonstrated a similar phenotype in plasmas A and B, but variable reactivity with plasma C. In contrast, ST5 clinical isolates frequently showed reactivity with plasmas D, G and H, which had showed almost no reactivity with ST8 isolates. Plasma samples C, D, H, and I were consistently reactive with ST30, ST36, and ST72 strains from a NARSA reference panel, while the ST45 and ST59 strains showed only φ< 0 in the entire panel.

**Conclusion:**

*S. aureus*-mediated fibrin formation is variable across strain and host. Within-ST patterns suggest that underlying bacterial genetic factors may determine staphylothrombin activity phenotypes in human hosts. Further understanding of these phenomena may lead to new ways to risk stratify and treat *S. aureus* bloodstream infections.

**Disclosures:**

All Authors: No reported disclosures

